# Supplementary material for: Pyruvate Dehydrogenase Kinase 1 inhibition mediated oxidative phosphorylation enhancement in cartilage promotes osteoarthritis progression
Source: BMC Musculoskelet Disord. 2023 Jul 20;24:597. doi: 10.1186/s12891-023-06585-6 (PMC10357736; doi:10.1186/s12891-023-06585-6)
Supplement: Supplementary file 1 — Additional file 1. [file 12891_2023_6585_MOESM1_ESM.zip › MF.Gsea.1653627316242/GOMF_METALLOENDOPEPTIDASE_ACTIVITY.html]

Details for gene set GOMF\_METALLOENDOPEPTIDASE\_ACTIVITY[GSEA]

|  || Dataset | 55457\_collapsed\_to\_symbols.cls.cls #OA\_versus\_normal.cls.cls #OA\_versus\_normal\_repos |
| Phenotype | cls.cls#OA\_versus\_normal\_repos |
| Upregulated in class | normal |
| GeneSet | GOMF\_METALLOENDOPEPTIDASE\_ACTIVITY |
| Enrichment Score (ES) | -0.3700195 |
| Normalized Enrichment Score (NES) | -1.5702363 |
| Nominal p-value | 0.004792332 |
| FDR q-value | 0.16076246 |
| FWER p-Value | 0.999 |
Table: GSEA Results Summary

  

Fig 1: Enrichment plot: GOMF\_METALLOENDOPEPTIDASE\_ACTIVITY      
 Profile of the Running ES Score & Positions of GeneSet Members on the Rank Ordered List

  

| SYMBOL | TITLE | RANK IN GENE LIST | RANK METRIC SCORE | RUNNING ES | CORE ENRICHMENT || 1 | ADAM7 | ADAM metallopeptidase domain 7 [Source:HGNC Symbol;Acc:HGNC:214] | 388 | 0.711 | -0.0017 | No |
| 2 | ZMPSTE24 | zinc metallopeptidase STE24 [Source:HGNC Symbol;Acc:HGNC:12877] | 451 | 0.682 | 0.0209 | No |
| 3 | ADAM28 | ADAM metallopeptidase domain 28 [Source:HGNC Symbol;Acc:HGNC:206] | 925 | 0.534 | 0.0055 | No |
| 4 | ECEL1 | endothelin converting enzyme like 1 [Source:HGNC Symbol;Acc:HGNC:3147] | 1704 | 0.398 | -0.0393 | No |
| 5 | CLCA2 | chloride channel accessory 2 [Source:HGNC Symbol;Acc:HGNC:2016] | 1787 | 0.386 | -0.0302 | No |
| 6 | CLCA4 | chloride channel accessory 4 [Source:HGNC Symbol;Acc:HGNC:2018] | 1967 | 0.364 | -0.0295 | No |
| 7 | ADAMTSL2 | ADAMTS like 2 [Source:HGNC Symbol;Acc:HGNC:14631] | 2185 | 0.340 | -0.0327 | No |
| 8 | ADAMDEC1 | ADAM like decysin 1 [Source:HGNC Symbol;Acc:HGNC:16299] | 2632 | 0.287 | -0.0560 | No |
| 9 | MEP1B | meprin A subunit beta [Source:HGNC Symbol;Acc:HGNC:7020] | 3061 | 0.244 | -0.0796 | No |
| 10 | MMP23B | matrix metallopeptidase 23B [Source:HGNC Symbol;Acc:HGNC:7171] | 3367 | 0.215 | -0.0948 | No |
| 11 | ADAMTS3 | ADAM metallopeptidase with thrombospondin type 1 motif 3 [Source:HGNC Symbol;Acc:HGNC:219] | 3468 | 0.205 | -0.0943 | No |
| 12 | ADAMTS2 | ADAM metallopeptidase with thrombospondin type 1 motif 2 [Source:HGNC Symbol;Acc:HGNC:218] | 3530 | 0.199 | -0.0911 | No |
| 13 | MMP1 | matrix metallopeptidase 1 [Source:HGNC Symbol;Acc:HGNC:7155] | 3625 | 0.191 | -0.0907 | No |
| 14 | UQCRC2 | ubiquinol-cytochrome c reductase core protein 2 [Source:HGNC Symbol;Acc:HGNC:12586] | 4020 | 0.154 | -0.1153 | No |
| 15 | ADAM9 | ADAM metallopeptidase domain 9 [Source:HGNC Symbol;Acc:HGNC:216] | 4171 | 0.140 | -0.1213 | No |
| 16 | COPS5 | COP9 signalosome subunit 5 [Source:HGNC Symbol;Acc:HGNC:2240] | 4261 | 0.131 | -0.1230 | No |
| 17 | MIPEP | mitochondrial intermediate peptidase [Source:HGNC Symbol;Acc:HGNC:7104] | 4278 | 0.130 | -0.1190 | No |
| 18 | ADAMTSL3 | ADAMTS like 3 [Source:HGNC Symbol;Acc:HGNC:14633] | 4421 | 0.119 | -0.1253 | No |
| 19 | MMP27 | matrix metallopeptidase 27 [Source:HGNC Symbol;Acc:HGNC:14250] | 4559 | 0.109 | -0.1316 | No |
| 20 | MMP3 | matrix metallopeptidase 3 [Source:HGNC Symbol;Acc:HGNC:7173] | 4719 | 0.094 | -0.1403 | No |
| 21 | THOP1 | thimet oligopeptidase 1 [Source:HGNC Symbol;Acc:HGNC:11793] | 5334 | 0.045 | -0.1864 | No |
| 22 | CLCA1 | chloride channel accessory 1 [Source:HGNC Symbol;Acc:HGNC:2015] | 5363 | 0.043 | -0.1868 | No |
| 23 | PMPCA | "peptidase, mitochondrial processing subunit alpha [Source:HGNC Symbol;Acc:HGNC:18667]" | 5454 | 0.037 | -0.1924 | No |
| 24 | MMP10 | matrix metallopeptidase 10 [Source:HGNC Symbol;Acc:HGNC:7156] | 5477 | 0.034 | -0.1927 | No |
| 25 | IDE | insulin degrading enzyme [Source:HGNC Symbol;Acc:HGNC:5381] | 5491 | 0.033 | -0.1924 | No |
| 26 | ADAM22 | ADAM metallopeptidase domain 22 [Source:HGNC Symbol;Acc:HGNC:201] | 5505 | 0.032 | -0.1922 | No |
| 27 | YME1L1 | YME1 like 1 ATPase [Source:HGNC Symbol;Acc:HGNC:12843] | 5528 | 0.030 | -0.1926 | No |
| 28 | PMPCB | "peptidase, mitochondrial processing subunit beta [Source:HGNC Symbol;Acc:HGNC:9119]" | 5630 | 0.022 | -0.1997 | No |
| 29 | MMP8 | matrix metallopeptidase 8 [Source:HGNC Symbol;Acc:HGNC:7175] | 5631 | 0.021 | -0.1988 | No |
| 30 | ADAM18 | ADAM metallopeptidase domain 18 [Source:HGNC Symbol;Acc:HGNC:196] | 5695 | 0.016 | -0.2031 | No |
| 31 | MMP26 | matrix metallopeptidase 26 [Source:HGNC Symbol;Acc:HGNC:14249] | 5749 | 0.012 | -0.2067 | No |
| 32 | NRDC | nardilysin convertase [Source:HGNC Symbol;Acc:HGNC:7995] | 5838 | 0.006 | -0.2134 | No |
| 33 | KEL | Kell metallo-endopeptidase (Kell blood group) [Source:HGNC Symbol;Acc:HGNC:6308] | 5912 | 0.001 | -0.2190 | No |
| 34 | MMP28 | matrix metallopeptidase 28 [Source:HGNC Symbol;Acc:HGNC:14366] | 5938 | -0.000 | -0.2209 | No |
| 35 | ADAM23 | ADAM metallopeptidase domain 23 [Source:HGNC Symbol;Acc:HGNC:202] | 6114 | -0.014 | -0.2341 | No |
| 36 | MMP20 | matrix metallopeptidase 20 [Source:HGNC Symbol;Acc:HGNC:7167] | 6623 | -0.052 | -0.2716 | No |
| 37 | MMP13 | matrix metallopeptidase 13 [Source:HGNC Symbol;Acc:HGNC:7159] | 6661 | -0.053 | -0.2724 | No |
| 38 | TMPRSS6 | transmembrane serine protease 6 [Source:HGNC Symbol;Acc:HGNC:16517] | 6841 | -0.066 | -0.2837 | No |
| 39 | MMP11 | matrix metallopeptidase 11 [Source:HGNC Symbol;Acc:HGNC:7157] | 6844 | -0.066 | -0.2812 | No |
| 40 | ADAM21 | ADAM metallopeptidase domain 21 [Source:HGNC Symbol;Acc:HGNC:200] | 7010 | -0.081 | -0.2908 | No |
| 41 | MMP2 | matrix metallopeptidase 2 [Source:HGNC Symbol;Acc:HGNC:7166] | 7037 | -0.082 | -0.2895 | No |
| 42 | ADAM12 | ADAM metallopeptidase domain 12 [Source:HGNC Symbol;Acc:HGNC:190] | 7727 | -0.136 | -0.3378 | No |
| 43 | MMP24 | matrix metallopeptidase 24 [Source:HGNC Symbol;Acc:HGNC:7172] | 7767 | -0.139 | -0.3353 | No |
| 44 | PAPPA | pappalysin 1 [Source:HGNC Symbol;Acc:HGNC:8602] | 7867 | -0.146 | -0.3371 | No |
| 45 | MEP1A | meprin A subunit alpha [Source:HGNC Symbol;Acc:HGNC:7015] | 7942 | -0.151 | -0.3369 | No |
| 46 | KLK7 | kallikrein related peptidase 7 [Source:HGNC Symbol;Acc:HGNC:6368] | 8053 | -0.159 | -0.3390 | No |
| 47 | ADAMTS13 | ADAM metallopeptidase with thrombospondin type 1 motif 13 [Source:HGNC Symbol;Acc:HGNC:1366] | 8451 | -0.190 | -0.3624 | Yes |
| 48 | ADAM29 | ADAM metallopeptidase domain 29 [Source:HGNC Symbol;Acc:HGNC:207] | 8462 | -0.191 | -0.3555 | Yes |
| 49 | PITRM1 | pitrilysin metallopeptidase 1 [Source:HGNC Symbol;Acc:HGNC:17663] | 8476 | -0.192 | -0.3488 | Yes |
| 50 | CLCA3P | "chloride channel accessory 3, pseudogene [Source:HGNC Symbol;Acc:HGNC:2017]" | 8517 | -0.195 | -0.3441 | Yes |
| 51 | MMP9 | matrix metallopeptidase 9 [Source:HGNC Symbol;Acc:HGNC:7176] | 8698 | -0.209 | -0.3497 | Yes |
| 52 | ADAM11 | ADAM metallopeptidase domain 11 [Source:HGNC Symbol;Acc:HGNC:189] | 8737 | -0.211 | -0.3442 | Yes |
| 53 | RCE1 | Ras converting CAAX endopeptidase 1 [Source:HGNC Symbol;Acc:HGNC:13721] | 8867 | -0.222 | -0.3453 | Yes |
| 54 | ADAM10 | ADAM metallopeptidase domain 10 [Source:HGNC Symbol;Acc:HGNC:188] | 8875 | -0.223 | -0.3369 | Yes |
| 55 | PRSS2 | serine protease 2 [Source:HGNC Symbol;Acc:HGNC:9483] | 8969 | -0.230 | -0.3349 | Yes |
| 56 | ADAM2 | ADAM metallopeptidase domain 2 [Source:HGNC Symbol;Acc:HGNC:198] | 8990 | -0.231 | -0.3272 | Yes |
| 57 | AFG3L2 | AFG3 like matrix AAA peptidase subunit 2 [Source:HGNC Symbol;Acc:HGNC:315] | 9045 | -0.235 | -0.3220 | Yes |
| 58 | ADAMTS5 | ADAM metallopeptidase with thrombospondin type 1 motif 5 [Source:HGNC Symbol;Acc:HGNC:221] | 9107 | -0.240 | -0.3171 | Yes |
| 59 | MMP12 | matrix metallopeptidase 12 [Source:HGNC Symbol;Acc:HGNC:7158] | 9285 | -0.254 | -0.3207 | Yes |
| 60 | MME | membrane metalloendopeptidase [Source:HGNC Symbol;Acc:HGNC:7154] | 9287 | -0.255 | -0.3105 | Yes |
| 61 | ADAMTS12 | ADAM metallopeptidase with thrombospondin type 1 motif 12 [Source:HGNC Symbol;Acc:HGNC:14605] | 9360 | -0.260 | -0.3057 | Yes |
| 62 | MMP17 | matrix metallopeptidase 17 [Source:HGNC Symbol;Acc:HGNC:7163] | 9369 | -0.261 | -0.2958 | Yes |
| 63 | ADAM30 | ADAM metallopeptidase domain 30 [Source:HGNC Symbol;Acc:HGNC:208] | 9809 | -0.302 | -0.3179 | Yes |
| 64 | PAPPA2 | pappalysin 2 [Source:HGNC Symbol;Acc:HGNC:14615] | 9868 | -0.309 | -0.3100 | Yes |
| 65 | ADAM20 | ADAM metallopeptidase domain 20 [Source:HGNC Symbol;Acc:HGNC:199] | 10028 | -0.324 | -0.3094 | Yes |
| 66 | ADAM8 | ADAM metallopeptidase domain 8 [Source:HGNC Symbol;Acc:HGNC:215] | 10054 | -0.326 | -0.2982 | Yes |
| 67 | TLL1 | tolloid like 1 [Source:HGNC Symbol;Acc:HGNC:11843] | 10235 | -0.345 | -0.2984 | Yes |
| 68 | MMP25 | matrix metallopeptidase 25 [Source:HGNC Symbol;Acc:HGNC:14246] | 10245 | -0.346 | -0.2852 | Yes |
| 69 | MMP19 | matrix metallopeptidase 19 [Source:HGNC Symbol;Acc:HGNC:7165] | 10652 | -0.388 | -0.3013 | Yes |
| 70 | TLL2 | tolloid like 2 [Source:HGNC Symbol;Acc:HGNC:11844] | 10823 | -0.407 | -0.2982 | Yes |
| 71 | ADAM19 | ADAM metallopeptidase domain 19 [Source:HGNC Symbol;Acc:HGNC:197] | 10838 | -0.409 | -0.2828 | Yes |
| 72 | ADAMTS8 | ADAM metallopeptidase with thrombospondin type 1 motif 8 [Source:HGNC Symbol;Acc:HGNC:224] | 10905 | -0.417 | -0.2712 | Yes |
| 73 | PHEX | phosphate regulating endopeptidase homolog X-linked [Source:HGNC Symbol;Acc:HGNC:8918] | 10992 | -0.429 | -0.2606 | Yes |
| 74 | MMP15 | matrix metallopeptidase 15 [Source:HGNC Symbol;Acc:HGNC:7161] | 11003 | -0.430 | -0.2441 | Yes |
| 75 | MMP7 | matrix metallopeptidase 7 [Source:HGNC Symbol;Acc:HGNC:7174] | 11073 | -0.441 | -0.2318 | Yes |
| 76 | BMP1 | bone morphogenetic protein 1 [Source:HGNC Symbol;Acc:HGNC:1067] | 11145 | -0.449 | -0.2193 | Yes |
| 77 | MBTPS2 | "membrane bound transcription factor peptidase, site 2 [Source:HGNC Symbol;Acc:HGNC:15455]" | 11280 | -0.468 | -0.2109 | Yes |
| 78 | ADAMTS20 | ADAM metallopeptidase with thrombospondin type 1 motif 20 [Source:HGNC Symbol;Acc:HGNC:17178] | 11337 | -0.476 | -0.1961 | Yes |
| 79 | MMP16 | matrix metallopeptidase 16 [Source:HGNC Symbol;Acc:HGNC:7162] | 11819 | -0.553 | -0.2114 | Yes |
| 80 | ADAMTS6 | ADAM metallopeptidase with thrombospondin type 1 motif 6 [Source:HGNC Symbol;Acc:HGNC:222] | 11845 | -0.558 | -0.1909 | Yes |
| 81 | ADAM17 | ADAM metallopeptidase domain 17 [Source:HGNC Symbol;Acc:HGNC:195] | 11931 | -0.579 | -0.1743 | Yes |
| 82 | SPG7 | "SPG7 matrix AAA peptidase subunit, paraplegin [Source:HGNC Symbol;Acc:HGNC:11237]" | 12020 | -0.597 | -0.1571 | Yes |
| 83 | MMP14 | matrix metallopeptidase 14 [Source:HGNC Symbol;Acc:HGNC:7160] | 12286 | -0.672 | -0.1508 | Yes |
| 84 | ECE2 | endothelin converting enzyme 2 [Source:HGNC Symbol;Acc:HGNC:13275] | 12319 | -0.682 | -0.1259 | Yes |
| 85 | ECE1 | endothelin converting enzyme 1 [Source:HGNC Symbol;Acc:HGNC:3146] | 12417 | -0.712 | -0.1048 | Yes |
| 86 | ADAMTS9 | ADAM metallopeptidase with thrombospondin type 1 motif 9 [Source:HGNC Symbol;Acc:HGNC:13202] | 12428 | -0.717 | -0.0767 | Yes |
| 87 | ADAMTS7 | ADAM metallopeptidase with thrombospondin type 1 motif 7 [Source:HGNC Symbol;Acc:HGNC:223] | 12466 | -0.733 | -0.0501 | Yes |
| 88 | ADAMTS1 | ADAM metallopeptidase with thrombospondin type 1 motif 1 [Source:HGNC Symbol;Acc:HGNC:217] | 12553 | -0.775 | -0.0257 | Yes |
| 89 | ADAM15 | ADAM metallopeptidase domain 15 [Source:HGNC Symbol;Acc:HGNC:193] | 12893 | -1.310 | 0.0005 | Yes |
Table: GSEA details [plain text format]

  

Fig 2: GOMF\_METALLOENDOPEPTIDASE\_ACTIVITY      
 Blue-Pink O' Gram in the Space of the Analyzed GeneSet

  

Fig 3: GOMF\_METALLOENDOPEPTIDASE\_ACTIVITY: Random ES distribution      
 Gene set null distribution of ES for **GOMF\_METALLOENDOPEPTIDASE\_ACTIVITY**
